# ‘Tipping the balance' – an evaluation of COVID-19 parenting resources developed and adapted for child protection during global emergency responses

**DOI:** 10.1080/21642850.2022.2104285

**Published:** 2022-08-04

**Authors:** Lorraine Sherr, Helen Mebrahtu, Kasonde Mwaba, Nisso Nurova, Angelique Nicole Chetty, Alison Swartz, Lucie Cluver, Kathryn J. Steventon Roberts, Jamie M. Lachman

**Affiliations:** a Institute for Global Health, University College London, London, UK; b Department of Social Policy & Intervention, University of Oxford, Oxford, UK; c Division of Social and Behavioural Sciences, University of Cape Town, Cape Town, South Africa; d Centre for Social Science Research, University of Cape Town, Cape Town, South Africa; e Department of Psychiatry and Mental Health, University of Cape Town, Cape Town, South Africa; f MRC/CSO Social and Public Health Sciences Unit, University of Glasgow, Glasgow, United Kingdom

**Keywords:** Parenting interventions, COVID-19, parenting challenges, children, parenting tips

## Abstract

**Background:** Parenting was severely affected by lockdown, school closure, illness, movement restrictions and the many sudden changes wrought by the global emergence of COVID-19. Responding to the need for a rapid emergency response to support parents and caregivers, a consortium of providers developed a suite of COVID-19 parenting resources based on evidence-based parenting interventions. Launched in March 2020, these were adapted for online use, with versions in over 100 languages, and the possibility for downloading, radio, and oral provision. A rapid qualitative evaluation initiative was conducted from September 2020 to February 2021 to inform the procedure, understand the impact and to drive future provision.

**Methods:** The evaluation collected openended responses surveys (n = 495 participants) and in-depth interviews with parents, providers, and adolescent children (n = 22) from 14 countries and one global source. Data were gathered on parenting challenges during COVID-19 and the utility of the COVID-19 parenting resources. In-depth, semi-structured interviews explored the same concepts and elaborated on challenges, utility of the resources, and recommendations for the future. Data were coded in a hierarchy from basic, organising and global theme generation.Results: The parenting resources equipped parents with information and practices transforming everyday lives, and interactions. The tips provided prompts and permissions related to children’s behaviour, enabled communications, and offered ways to reduce stress, monitor behaviour and navigate discipline challenges. The timeliness of the resources as well as the clarity and ease of use were seen as advantages. Future direction and possible hurdles related to adaptations needed according to recipient, child age, local context, culture, and new challenges.

**Conclusions:** Overall findings point to the value and utility of this unprecedented global response to theCOVID-19 pandemic. Results suggest that rapid provision of parenting resources at scale is feasible and of use and opens a pathway for providing evidence-based interventions under COVID-19 constraints.

**CONTRIBUTIONS TO THE LITERATURE**

- The findings of this study inform the literature on the challenges faced by parents globally, during the COVID-19 pandemic, using qualitative research methods
- Our data shows that rapid provision of parenting resources at scale is feasible under COVID-19 constraints and beneficial for parents across different settings
- The results also highlight implications for the development of future COVID-19 parenting resources and demonstrate the urgent need for more family-based interventions to support parents during global pandemics

## Introduction

The COVID-19 pandemic has created new challenges for families and children (UNICEF Beyond Masks, 2021) through economic and healthcare disruptions, limiting social networks, restricted mobility, and school closures (Cluver et al., 2020). The United Nations Educational, Scientific and Cultural Organisation further estimates 1.38 billion children are cycling in and out of school or childcare, without access to group activities, team sports, or playgrounds (Perks & Cluver, 2020). While the full implications of the COVID-19 pandemic on child wellbeing are still unfolding, emerging evidence suggests that the pandemic is exacerbating vulnerabilities to negative child outcomes in already disadvantaged and marginalised families and children (United Nations Office of Research- Innocenti, 2020). These factors have serious implications such as on mental health distress, abuse, parental stress, and violence against children (Unicef UNICEF Office of Research – Innocenti, 2020). It is understandable that the challenges parents are facing under the strain of the pandemic are near-universal, and most harsh parenting might not be malicious, but rather triggered by stress, poverty, and mental health distress (Skar, Sherr, Macedo, Tetzchner, & Fostervold, 2021). However, in the extreme cases, where violence and neglect has worsened, those perpetuating abuse may have had increased impunity and victims have been cut off from supportive teachers, social workers, and friends. In these challenging environments, the looming economic fallout and uncertainty is adding yet more pressure onto such family settings, with lifetime and intergenerational consequences for the children affected (Chen & Chan, 2016).

Global research tells us that parenting behaviour can have an immediate and long-term impact on child health and developmental outcomes in the long run (Sherr et al., 2017). There is also sound evidence that indicates that good parenting is beneficial (Sherr, Skar, Clucas, von Tetzchner, & Hundeide, 2014) and associated with enhanced child self-esteem, educational attainment and reduced behavioural problems, depression, and trauma (Skar, von Tetzchner, Clucas, & Sherr, 2015). There are several evidence-based interventions that have been shown to improve parenting (Knerr, Gardner, & Cluver, 2013), and thereby improve child wellbeing and reduce violence against children (McCoy, Melendez-Torres, & Gardner, 2020) during emergency contexts, such as the COVID-19 pandemic. Parenting programmes have also been shown to improve parent–child relationships, reduce caregiver stress and violence during discipline (Coore Desai, Reece, & Shakespeare-Pellington, 2017), as well as improve child and caregiver mental health (Chen & Chan, 2016), in low middle- and high-income settings (Skar et al., 2015). Such evidence-based parenting programmes support families with the common challenges of raising children while respecting parents’ capacity to solve problems. They also provide effective strategies for improving relationships, reducing conflict, managing family finances, and relieving parenting stress (Ward et al., 2020). Results from randomised controlled trials have shown that families accessing parenting programmes have reductions in violence, mental health problems, alcohol use and extreme poverty (Cluver et al., 2018). There is also some evidence of reduced abuse reported by both caregivers and adolescents, as well as improvements in parenting and parental supervision, improvements in household economic welfare and financial management (Puffer et al., 2017). Other interventions have shown that violence can be halved by combining parenting with cash transfer programmes, community mobilisation and education (Meink Meinck et al., 2019). Successful interventions include responding to online protection risks and using multimedia messaging such as through radio and social media campaigns to raise awareness about resources for support (UNICEF Beyond Masks, n.d). These evidence-based interventions have demonstrated that through adaptation, they can mitigate adverse impacts in emergency settings, such as the COVID-19 pandemic.

Although there is good evidence in the literature that show parenting interventions may be effective public health approaches to reduce child maltreatment, there is a need for a universal, public health approach to reduce the impact of COVID-19 in addition to delivering these interventions at scale. The context for delivering these interventions has changed and continues to change because of COVID-19, thus requiring further adaptation of interventions to address the need for physical and social distancing, movement restrictions and weakened government capacity^1^. The COVID-19 Playful Parenting Emergency Response initiative began on 23 March 2020, when a billion children were taken out of school by global lockdowns. Parenting for Lifelong Health alongside partners (including the WHO, UNICEF, World Without Orphans, World Childhood Foundation, Internet of Good Things, University of Oxford, United Nations Office on Drugs and Crime, IDEMS, Maestral, Together for Girls, the Global Partnership to End Violence, UKRI GCRF Accelerate Hub, USAID, UCL and the US Centers for Disease Control and Prevention) has led an urgent response to develop open-source COVID-19 parenting resources to support parents and caregivers during the COVID-19 pandemic and beyond. This group converted the Parenting for Lifelong Health programmes – evidence-based open-source parenting programmes tested in ten randomised trials – into simple tips sheets for parents, radio scripts, and public service announcements, and other modalities for population-level dissemination. These were immediately translated into over 100 languages by global volunteers, led by a faith-based network World Without Orphans, made open source on a website www.covid19parenting.com, and launched in a Lancet letter (Cluver et al., 2020).

**Figure 1:**
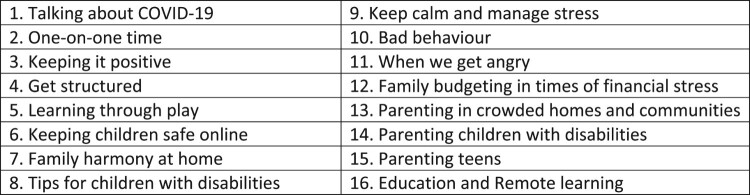
. Covid-19 Parenting tips

The COVID-19 parenting resources aim to support positive parent–child relationships and reduce violence against children. They consist of a set of 16 topics, including learning through play, reinforcing positive behaviours, managing difficult behaviours, creating structure and routines, talking about COVID-19, keeping children safe online, and reducing stress and conflict (see Figure 1). The resources also include specific content for parents of adolescents, infants, and toddlers, and those living with disabilities. Resources have been adapted for a variety of delivery platforms including radio, sermons, social media, public service announcements, caseworker and home visitor guides, telephone consultations, and animated videos. In collaboration with agencies and governments disseminating these resources globally, these parenting tips have reached a minimum of 136 million families by January 2021. Understanding the effectiveness, utility and experience of the individuals utilising these resources is critical for evaluation, and to inform similar future programming responses, should they be required.

### Study aim

This study was conducted from September 2020 to February 2021 and utilised qualitative methodology to provide in-depth insight into parenting challenges during the COVID-19 pandemic and the practical utility of the COVID-19 parenting resources among caregivers of children across different global settings. This study was implemented alongside a quantitative study examining the resources’ reach, effectiveness, adoption, implementation, and maintenance of the resources, and aims to expand such data to provide rich detail on the opinions, realities and experiences of individuals parenting in the time of a global pandemic as well as their access to and utility of accessing and utilising.

## Methods

### Study design

Qualitative insights into the COVID-19 parenting tips were explored retrospectively in two ways: **1)** by adding open-ended questions to a series of quantitative surveys, **2)** conducting in-depth interviews with parents, adolescents, and implementers (utilising online software to adhere to social distancing protocols). Participants either completed the open-ended question or the in-depth interviews. Methodology was country specific, hence country data is either drawn from open-ended questions or in-depth interviews.

### Open questions within quantitative surveys (parenting challenges and utility of the COVID-19 parenting tips)

To generate the respondents targeted purposive sampling was used to identify partner organisations who were disseminating or planning to disseminate parenting resources, and who would be willing to participate in the evaluation initiative. Retrospective and pre–post survey questionnaires were distributed through these organisations. These quantitative surveys included two open-ended questions: ‘*Describe parenting challenges you have experienced during the COVID-19 pandemic,*’ and ‘*How did you use the parenting tips?*’. The responses were anonymised with only country, gender, and age of respondent available for analysis. Only responses of more than a single word were eligible for inclusion in the analysis. All responses to the open questions were collated, translated to English, and analysed thematically. Respondents were international and drawn from six countries (Paraguay, Malawi, Nepal, Ghana, North Macedonia, Cameroon).

The thematic analysis of open questions involved a process of the stepwise familiarisation with the data (Nowell, Norris, White, & Moules, 2017), generation of initial codes, interrogation of these for themes, and then theme review (Braun & Clarke, 2006). Once this was completed, labels and definitions for coding themes were generated. These were then classified and consolidated into higher order and macro themes. The analysis plan included an additional step of carrying out content analysis on the emerging themes. Two independent researchers (supervised by a third researcher) coded the survey questions. Disagreements in coding were resolved by team discussion. The average length of open-ended responses was 15.4 words (mean; range 1–125 words [based on English translations]).

### In-depth interviews (impact of COVID-19 parenting tips on users)

From October to December 2020, participants were recruited for interviews using purposive sampling and snowballing techniques. In-depth interviews (n = 22) were conducted with adolescents, parents, and organisation staff (facilitators) online using Zoom software according to a semi-structured interview protocol.

Interviews included facilitators with an international perspective as well as respondents from eight countries (see Table 1). A purposive sample of 22 in-depth qualitative interviews were conducted with implementing agencies who were the facilitators in dissemination (named facilitators in the report; n =  7), parents/caregivers (n = 14) and adolescents (n = 3) whose families were engaged with the tools. Respondents were international and drawn from eight countries (United Kingdom, USA, South Africa, Zimbabwe, Israel, Sri Lanka, Pakistan, India: n = 1-2 interviews were conducted per country, with the exception of South Africa [n = 11]). Interviews were conducted in English by a trained researcher (n = 20), or in local language by a trained partner (n = 2), transcribed and translated and into English where necessary. Four researchers were involved in the data collection, coding, and analysis process. The first researcher recruited participants and conducted the interviews in English (n = 20). A second trained facilitator conducted two shortened interviews in local languages, recorded these verbatim, translated to English, and added to the interview database. Two independent researchers coded interviews. Interviews explored participant exposure to and utilisation/experience of the parenting tips together with recommendations for improving the resource. The interview protocol covered core elements with slight adjustments depending on the recipient and their use of the COVID-19 parenting tips including organisations, parents/caregivers and adolescents. The average length of interviews was 22.3 min (range 7–46 min).

**Table 1:** . Breakdown of responses collected from the online survey

Data source	Open questions pre	Open questions post	Depth Interviews	Total Usable
Open Questions				
Paraguay	275	90		302
Malawi		27		26
Nepal		8		8
Ghana		18		16
North Macedonia		38		31
Cameroon		112		110
Global –online source		3		2
In-depth Interviews				
International			2	2
United Kingdom			2	2
USA			1	1
South Africa			11	11
Zimbabwe			2	2
Israel			1	1
Sri Lanka			1	1
Pakistan			1	1
India			1	1
TOTAL	275	296	22	517

### Eligibility criteria for participant inclusion

The following are the inclusion criteria for pre–post surveys and qualitative data collection with participating parents or caregivers (n = 30-200 per country):

1. Any person caring for a child up to 17 years
2. Has received or will receive COVID-19 playful parenting tips
3. Has provided consent and/or assent for child to participate in the study.

### Data analyses

Given the differences within the data sources of this study (open questions and in-depth interview data) it was decided that analyses would utilise a combination of content analysis and thematic network analysis as described by Attride-Stirling (2001) (Attride-Stirling, 2001). Due to the nature of the data from the open-ended questions, content analysis was deemed an appropriate methodology, and thematic network analysis was utilised due to its systematic approach to analysis, allowing for clarity in the development of arguments in relation to a particular phenomenon. The open questions were translated to English and entered into an Excel spreadsheet for analysis. The interviews were transcribed automatically using Zoom’s audio transcription feature. The resulting transcripts were then formatted and checked for accuracy to ensure quality and entered into Nvivo 12 for analysis. Participants’ identifiable information was removed, and each assigned a participant ID number.

The analysis process involved the identification of three levels of themes (basic, organising, and global) generated from responses to the open survey questions (495 responses from pre and post open questions; see Table 1). Data was coded into ‘manageable and meaningful’ segments (Attride-Stirling, 2001) guided by the core issues of interest of the study e.g. challenges of parenting under COVID-19 and evaluation of the COVID-19 parenting tips including their utility and applicability. The resulting codes were then reviewed and grouped together into basic themes. These themes centred on the coverage of similar topics or ideas. Content analysis was used to enumerate the frequency of themes to explore their representativeness across the sample. Utilising these analyses, themes were then grouped together into organising themes and then global themes, which illustrated the overarching patterns in the data as a whole. The resulting themes were then arranged into thematic networks, web-like structures depicting the relationships between the basic, organising, and global themes. Four independent researchers were involved in data collection and the coding of themes. The data collection, coding, analysis, and reporting of findings was supervised by a senior researcher/supervisor. Illustrative quotes are set out for the various themes, with identifiers to demonstrate reporting across the breadth of the sample. Identified themes are presented in the results section (below).

## Results

A total sample of 593 open-ended survey responses were gathered, with 517 responses eligible for inclusion. Seventy-six single-word responses to open survey questions were excluded as they could not be accurately interpreted or coded. The in-depth data base included 240 responses to open survey questions gathered pre-exposure to the COVID-19 parenting tips: 255 open question responses from those post-exposure to the tips, and 22 in-depth interviews. Table 1 sets out the data sources and the total usable responses. In total the data were gathered from 14 countries as well as global online and international groups (see Table 1).

### Emerging themes

The overall analysis generated one global theme relating to parenting challenges, with nine organising sub-themes, and four global themes related to parents’ perception of the utility of the parenting resources, with twelve organising sub-themes.

### Parenting challenges

The first global theme explored the challenges parents experienced during the COVID-19 pandemic (see Table 2 for summary with each organising theme and extant quotes). Parents reported grappling with difficult situations that they had not envisioned such as keeping children busy and entertained during the lockdown, navigating heightened emotions, restrictions on their everyday lives, juggling their personal and professional lives, and ensuring the wellbeing of their families.

**Table 2:** . Thematic breakdown of comments and example quotes from participants for parenting challenged faced

Global theme	Organising themes	Selected quotes
COVID-19 pandemic induced diverse and complex parenting challenges	Keeping them busy and entertained – *Innovation lab*	*‘Finding different activities to avoid boredom during their free time’ (Parent/Male/Paraguay)*
	Communication – *Prompts and permissions*	*‘To get through 24 h with a badly mannered child and not to scream or hit her.’ (Parent/Female/Paraguay)* ‘*I like it a lot and lots of the tips, I do. Other things I knew and had forgotten, so I remembered.’ (Parent/Female/Paraguay)*
	Discipline – *Order and boundaries*	*‘Playing a lot with my children and lots of disciplining even when playing and teaching them to be responsible, even when playing’ (Parent/Male/Paraguay)*
	Balancing parenting with other responsibilities (including childcare) – *'Juggling roles'*	*‘When COVID came I started working more from home and that you know that changes things cause I mean when you're home, she's expecting attention so it now means balancing giving her time when she woke up, and usually that will be as scribbling on paper. It was very difficult as a parent. Your child is seeing you at home. But you are working And, you know, you're still expected to be working and you're still expected to have calls but then little girl has no idea what that means, this little girl sees mommy around and wants to access mom's room to be able to play or talk to mom and moms closing the door moms like no you can't come in and she's like, why, you know, so I'd have moments where she's banging on the door saying mommy and crying. And wanting access and me depriving us of that. So it really became difficult like it was difficult at first for me’ (Parent/Female/32/Zimbabwe)*
	Teaching/virtual classes- ‘*School in a box’*	*‘So we went into full lockdown from around about the 26th of March. … . So school, became online and it was a matter of actually trying to find a quiet space in the house, for each child to be able to do their work. And set up a little almost classroom in each different room when they couldn't be disturbed during the day’ (Parent/Female/40/South Africa)*
	Time – '*The longest day'*	*‘Paying attention all the time, for what they need of my time, all the time.’ (Parent/Female/Paraguay)*
	Mental Health challenges/ Psychological stress- ‘*All in the mind’*	*‘the environment was this sort of very much an unknown environment. We didn't know when it was all going to end. We didn't know what the way out was. There was a lot of background fear the news was not lighthearted. It was ‘my God we're all going to die of COVID’ - so we were in a stressful environment. And so, for us as parents, we both work full time and then suddenly having three children in the house for the entire day was quite stressful’ (Parent/Male/41/UK)*
	Safety	*‘As I have 4 minors, the challenge was for them to feel safe, responsible with their tasks of school ensuring a habitual time and space to do them … ’(Parent/Female/Paraguay)*
	COVID-19 lockdown restrictions	*‘I live in a shared space at home and I'm the only one was working. How am I going to do this? How am I going to support everyone and still keep my baby safe and for me, it felt like it was the end of the world and at the peak of my career … .. We can't buy anything I can't and then everyone was talking about it like everyone was talking about. Have you stocked enough food? Have you stocked sanitisers? Have you stocked all those things to protect your home and everything and I have none of those I had not planned for all of those things I myself, what's going to happen now and I'm under budget for all of them because they were not on the list. And it was all of these feelings happening and I was really freaking out. I was very overwhelmed’ (Parent/Female/28/South Africa)*

Generally, parents described challenges with establishing new routines in light of the changes brought on by the pandemic. They also described challenges in maintaining established routines with their children, e.g. with bedtime routine. Challenges of keeping children of different ages busy and engaged in different activities while living under lockdown restrictions was another concern reported by parents. Additionally, physical wellbeing and mental health concerns, restricting child use of social media/virtual technology, and challenges in home schooling, were detailed. Furthermore, parents described challenges with dealing with the emotional stress brought on by the COVID-19 pandemic. They described anxiety related to the uncertainty of the pandemic and dealing with restrictions and lockdowns as stressful, especially when their children were not in school.

Balancing working from home and caring for their children was another major difficulty that parents highlighted. They described trying to strike a balance between the two as very difficult, and another significant daily stressor. Parents described additional stress associated with COVID-19 restrictions, as they prevented them from engaging in activities, they usually engaged it. They described engaging in mundane tasks that are usually taken for granted as becoming risky.

### Utility of COVID-19 parenting tips

**Four global themes** relating to user perception of the utilisation and evaluation of the COVID-19 parenting tips were identified (see Table 3). These themes reflected the applicability, utility, reach, and facilitators and barriers to the uptake and implementation of the parenting tips from various stakeholders (parents, facilitators, adolescents) that participated in this qualitative study.

**Table 3:** . Thematic breakdown of comments and example quotes relating to utility of COVID-19 parenting tips from participants

Global theme	Organising themes	Selected quotes
Parenting tips equipped parents with important information and practices that transfromed their everyday lives	Developing new parenting skills	*‘All tips for positive parenting were welcome. In times of crisis, somehow it is easier to get out of control and forget even those good parenting skills that we already have. What was new I certainly tried to change. Many tools calmed the domestic situation.’ (Parent/Female/North Macedonia)*
	Impact on behaviour	*‘It's like I never knew how to parent. I could easily get disappointed, and charge at my children. As a result, they were failing to express themselves. But now, it is just coming naturally, the smiles and laughter- and a number of people have admired my style of parenting. To me, it has been this idea of not just reading them, but being able to see- one by one, how the tips are making a difference in the life of my family. I really needed these tips.’ (Parent/Female/Malawi)*
	Violence reduction	*‘No more stress, hitting and spanking my children because of use of the tips. I now enjoy my children and plan activities with them’ (Parent/Female/Cameroon)*
	Improved communication	*‘The tips have acted like medicine to me as am not shouting or screaming at my children. They now do their work well in the house.’ (Parent/Female/Cameroon)*
	Improve wellbeing and reduce mental health burden	*‘It is a joy to witness such an amazing weight of stress these tips have removed off my shoulders as I parent. For me, it has been very engaging, and also challenging others, especially young couple friends to follow suit. It is very sustainable and doesn't require such formality to deliver.’ (Parent/Male/Malawi)*
	Ticking time	*‘I spend a lot of time with them. I listen to them carefully. We discuss anything in a deep and subtle way.’ (Parent/Female/Nepal)*
		
Construction of parenting tips resource facilitated uptake	Accessible	*‘Well, I love the visual, just looking at the things that looks really engaging and reports like kind of these clouds, which are inviting to read in a time that's particularly stressful. It looks, positive, the bright colours are kind of easy to follow. I like the look of them’ (Parent/Female/30/Israel)*
	Practical	*‘ … what I really liked about the tips. Is it had all the different ages. And I like the way that it just sort of focus with a practical methodology way of doing it’ (Parent/Male/41/UK)*
		
Challenges to implementation	Barriers to upake	*‘I think there's a lot of advice on what to do with teenagers and older children and not so much for toddlers and they're like the fact that it's just two babies and toddlers there’ (Parent/Female/32/Zimbabwe)*
	Barriers to dissemination	*‘It was my responsibility to plan this and make sure that the implementation is exactly how it was planned it was a bit tricky for me because something like this. I had undertaken this for the very first thing, so I didn't know how it was going to happen. And because I'm at my home, which is like an hour and a half from where the sessions were conducted and because I can't go there because of the situation outside It was even more difficult because I'm planning everything and then I have to implement and make sure that it's happening properly. I have to supervise that but I'm doing everything from home.' (Facilitator/Female/India)*
		
Creation of an enabling environment facilitated dissemination	Shared vision	*‘ … it's because I think everybody who we partnered with had a common vision common purpose. It, it wasn't something that we had to strive for’ (Facilitator/Male/Sri Lanka)*
	Wide-scale collaboration	*‘I think one positive thing that I appreciated was allowing people to speak into your process to say here could you try this out. And then that feedback is included into the process that I appreciated that.’ (Facilitator/Male/Malawi, Zambia, Zimbabwe)*

Parents and facilitators also reported perceived benefits of using the parenting resources. Parents indicated learning practical skills to improve their parenting techniques after using these resources. The resources helped parents learn new ways of dealing with challenges and they used them as a daily guide for problem solving as well as redirecting negative behaviours. Parents highlighted how the resources have greatly improved communication between themselves and their children. They started to listen and engage with children in more discussion as a family. The parenting resources also provided information about COVID-19, which were described by parents as facilitating conversations with children about COVID-19. The resources were also described by parents, adolescents, and facilitators as increasing knowledge about COVID-19 among individuals and within the communities they live and/or serve. Parents further described the parenting tips as a good resource for ideas of activities to keep their children engaged, especially since they were spending so much time indoors.

Of importance, parents disclosed that as a result of engaging with the parenting tips, their use of harsh or physical disciplining when children misbehaved had declined. This was perceived to have improved their relationship with their children and reduced the stress of parenting difficult children. Also, parents described the parenting resources as instrumental in raising awareness of ‘quality’ time and the importance of ‘one-on-one’ time with children.

Furthermore, parents and facilitators described the parenting resources as visually appealing, well-designed, and engaging, and repeatedly emphasised its bright colours. Facilitators and parents also described the resources as easily understandable and digestible. It is the simplicity of the resources that facilitated their wide-scale implementation and utility by different beneficiaries. At the same time, respondents noted challenges and barriers to the implementation of the resources. The online availability of the parenting resources as pdfs was described as limiting the accessibility by both parents and facilitators due to limited or lack of internet and computing resources in some contexts. Participants noted that some of the parenting tips were not applicable to the age of their child (specifically there was a desire for more resources for younger children) and this hindered their ability to use the resources. Additional prominent barriers to the usage and uptake of the parenting resources for caregivers included individual and cultural differences regarding parenting practices (compared to the parenting resources). For facilitators, prominent themes relating to barriers to uptake included difficulties in the translation process required to disseminate resources at a global scale and lockdown restriction. However, the creation of an enabling environment such as having a shared vision or common goal and wide-scale collaboration with multiple partners involved in the project, were found to be key in facilitating the timely dissemination of the parenting tips.

## Discussion

This study utilised qualitative methodologies to explore the perceptions and experiences of parents, adolescents, and facilitators with parenting challenges in the COVID-19 pandemic, as well as the utility of the COVID-19 parenting tips. Within the exploration of parenting challenges, ten themes were identified. Themes explored challenges with entertaining children, communication, discipline, balancing parenting responsibilities, education, mental health, safety, and the impacts of lockdown restrictions. Within the exploration of the utility of the COVID-19 parenting tips – four global themes emerged focusing on participant perceptions of the tips, their application and/or implementation, facilitators and barriers and recommendations for improving the resource. Findings demonstrate the benefits of the COVID-19 parenting resources as well as their acceptability and utility and highlight the need for and benefit of family-based interventions to support parents during this pandemic.

The COVID-19 pandemic disrupted the lives of millions of individuals and families around the world (Cluver et al., 2020; Lawson, Piel, & Simon, 2020; Wardell et al., 2020). These disruptions (e.g. school closures, restrictions on movement and social interactions, employment) and restrictions placed to reduce the spread of the virus, have had and will continue to have significant negative consequences on the wellbeing of populations (Cameron et al., 2020a; Chu, Schwartz, Towner, Kasparian, & Callaghan, 2020), and how they make meaning of the experience (Todorova et al., 2021). Parents are experiencing increased stress as a result of the pandemic and negotiating its consequences on everyday life. Findings form this study support such notions, specific stressors that were widely described by participants included emotional stress (Brown, Doom, Lechuga-PENA, Watamura, & Koppels, 2020a; Goldberg, Mccormick, & Virginia, 2020; Weaver & Swank, 2020), balancing work with parenting (Griffith, 2020), assisting children with schooling from home, keeping children occupied (Manohar et al., 2020), providing for families (Jenco, 2020), low levels and/or lack of social support, and restrictions on activities/movement. These stressors can profoundly affect the health and wellbeing of parents and increase the risk of parental burnout which may lead to child maltreatment and/or abuse. Challenges experienced by adolescent participants revolved around being unable to do what they usually do, including attending school, socialising with their friends and boredom – core challenged for adolescents which may have implications for mental health and wellbeing. Yet, a prominent theme emerging focused on adaptation and creativity highlighting possible resilience among adolescents. Findings highlight some of the core challenges for parenting during the pandemic, supporting previous literature and provide a foundation for future studies exploring such challenges. However, the longitudinal impacts of such challenges remain unexplored.

***Filling a gap:*** For many respondents, the resources were not by any means their only source of support but filled a gap or void. They were perceived as a welcomed additional source of support. They served to remind parents that experiences were shared, reactions were common, and small changes and tweaks could have considerable benefit. In terms of reducing frustration, harsh reactions, and violence, many felt the tips were particularly helpful by providing ideas on control, pausing, thinking, and introducing routines.

***Design and dissemination:*** There were various aspects of the design and dissemination process attributed to the success of the parenting resources by participants. The parenting tips were found to be widely acceptable, with participants having emphasised acceptability of the design and presentation of the information, which made them practical and accessible. The timeliness of the resources was perceived as a significant factor, as they were released early in the pandemic which allowed parents in need to have quick access to support and facilitators. Their grounding in the research literature increased their credibility and their universal approach encouraged uptake in a variety of contexts. The visually appealing nature of the tips and their simple language made them attractive and engaging to a wide audience, facilitating their application. The online format of the resources increased the reach as it was available anywhere to anyone with access to the internet. Given such versatility, extended reach was enabled. The tips were also applicable to children of various ages, which increased the utility and applicability of the tips. However, there was some discussion about age specific and age relevant input which may need consideration if the tips are to be expanded further.

***Collaboration:*** The successful dissemination of the parenting tips was attributed to the skilful collaboration of a diverse group of individuals and organisations with a shared vision to help parents address parental challenges and safeguard the wellbeing of children. Having shared organisational interests/activities and good working relationships allowed partners to work cooperatively. The involvement of a variety of actors and organisations at the global, national, and local level supported efforts to reach large numbers of individuals with the parenting tips. Encouragement of local involvement in adaptation of the resources, promoted ownership and allowed the resource to be disseminated in various formats, including posters and pamphlets to reach individuals who may not have had the opportunity to access the tips. The successful dissemination of the tips highlights the implications of choosing intervention partners wisely, especially in resource limited settings.

***Evidence-based:*** The solid evidence base underpinning the tips was mentioned and seen as a key building block for the resource. Even though the content was straightforward, accessible, and easy to follow some parents saw the tips as prompts and permissions to parent their child. It reminded participants of activities they had used before and helped with ideas and inspiration in times of great uncertainty. For some the information was new, for others a reminder. This clearly underpins the notion that parenting can be learned, taught, adapted, and continuously improved.

***Cultural and social context:*** The dissemination process was not without its challenges. Participants described some barriers to the utilization of the tips such as its online format which limits access for those without internet. This highlights the importance of developing resources in various formats, especially when trying to reach marginalised populations. Cultural factors, including language and local social norms related to parenting, could also act as barriers to the uptake of these parenting tips. Working with local communities to adapt the parenting tips to the context, increases their acceptability, and allows for local cultural and local social needs to be engaged without affecting the essence of the evidence-based messages. The fact that resources were free and open access was an important facilitator for immediate adoption and utilisation.

***COVID-19-related constraints:*** Although the content was derived from evidence-based programmes, the nature of the pandemic and the COVID-19 tips meant that group interactions, peer-learning and skill building with a leader present in other parenting programmes were not possible to replicate in the online and virtual forms. Some of the participants reflected on the need for such interactive activities and suggested ways of enhancing the shared experience with additions such as personal stories, ideas or tried solutions or some form of virtual dialogue through chat or interactions – suggesting considerations for future development.

***Future development:*** Discussions about further development of these parenting resource heavily focused on developing additional content parents would like to see covered. Participants described a wide variety of topics they would like to see covered, and audiences to be targeted. The content suggestions illustrate the growing information needs and challenges around returning to school, vaccination issues and long-term effects faced by parents that need to be addressed. It is necessary that the tips are revised to cater to various parenting roles, as they were seen as targeting mothers and therefore making gendered assumptions about parenting responsibilities. Analyses from the retrospective and pre–post quantitative data collection may also provide further insights into the impact of the resources in each of the case studies as well as overall.

**Figure 2:**
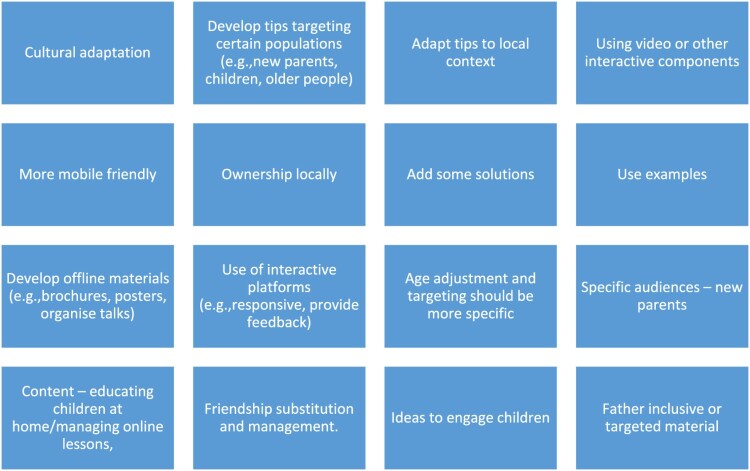
. Recommendations for further resource development

***Future recommendations:*** Recommendations also surrounded modes of delivery of parenting information, which focused on the use of more offline, accessible, and interactive methods. The use of other offline methods, such as posters and face-to-face presentations would help avoid some of the barriers discussed earlier concerning internet access. The use of more accessible formats such as audio, video and a more mobile friendly design would make the tips more accessible to certain populations and potentially engage more individuals who favour such modalities. The desire for interactive platforms to get parenting advice, highlights a need for a higher level of interaction that provides feedback. Use of such platforms could be an especially helpful source of support for parents who may be feeling isolated and/or unable to access their usual sources of support. Some consideration should be given to who plays a central role in parenting in different contexts, including mothers, fathers, grandparents, siblings and other caregivers. Lastly, recommendations concerning the dissemination process emphasised the need to create organised and efficient translation processes and engage more partners to expand the reach of the materials, especially within countries. Alternative modes of delivery were also important (such as radio and print versions handed out with food parcels) as many people do not have ready access to online resources and the digital divide is a challenge for the future. Figure 2 below highlights important recommendations for further development of the parenting tips resource.

### Limitations

Studies have recognised the need for evidence-based interventions such as this one to support parents during this extremely difficult time (Cameron et al., 2020b). The COVID-19 parenting tips addressed the needs of parents through providing advice for dealing with common challenges brought by the COVID-19 pandemic (Brown et al., 2020b). This study has a few limitations, including its cross-sectional nature, possibility that the experiences of reported challenges are changing due to the evolving nature of the COVID-19 situation especially in different contexts, which might limit the generalisability of the findings beyond the time of data collection, and social desirability bias. As this was a post evaluation of resources that were globally disseminated and widely taken up, we only received pre-evaluation data from one country (Paraguay), thus the views on parenting challenges might be underrepresented. Likewise, while in-depth interviews were undertaken in eight countries, for most countries only 1–2 interviews were conducted. Given this, the views presented within in-depth interviews may not be wholly representative of the broader population experience. Variations in global responses were not captured fully in this evaluation, yet the resource was globally downloaded, translated into over 100 languages and thus seemed to reach levels of acceptability. More specific data would enhance the understanding for different groups. Nevertheless, the findings from this study demonstrate the utility of the parenting tips to improve parenting experiences during the COVID-19 pandemic and beyond.

## Conclusion

The results of this study inform the literature on the challenges faced by parents during the COVID-19 pandemic using qualitative methods. The data highlights implications for the development of future COVID-19 parenting resources and demonstrates the urgent need for more family-based interventions to support parents during this pandemic. This glimpse into the experiences and utility of the parenting tips serves to underscore the specific challenges that many faced with sudden onset and with little recourse to support. The importance of evidence-based interventions is clear, and the timelines and rapid uptake of these resources could not have been made available if there had not been thorough trials and evaluation in the past. The pandemic has put parenting under pressure, but also provides an opportunity for evidence-based interventions to become rapidly available and utilised. These lessons could endure over the course of the pandemic and beyond. To date the tips have been downloaded by close on 200 million readers and the advent of the Ukrainian war has prompted a new opportunity for adapted provision.

In conclusion, the COVID-19 parenting tips have been demonstrated to be relevant, highly acceptable, useful, and versatile, which increases their utility and potential to reach many individuals and communities. The absence of negative comments or feedback with regards to the parenting tips from both data sources collected in this study is a key finding. Suggesting that such input is welcome when parenting during the pandemic, and attention to parenting is needed, desired, endorsed, and urgent.

## Declarations

### Author contributors

The qualitative study was led by LS, HM, and KW. All authors were involved in the setting up of the study, interpretation of the analysis, and manuscript writing and reviewing. JML, LC,LS contributed to the initial resources and led the overall evaluation process. LS, HM and KM took the lead on the qualitative data, analysis and coding, supported by KM, NN, ANC and AS. The write up was drafted by LS, HM, and KW, with full comments and additions by the team. All authors read and approved the final manuscript.

### Ethics statement

Ethical clearance for the evaluation of this work was granted by Oxford University (REF No R69569/RE002). In relation to adherence to the ethical requirements of the University of Oxford, this work complies with Approved Procedure 4 (AP4) that outlines how to manage the use of questionnaires or interviews that may include questions about sensitive topics. All data collection was carried out during the COVID-19 pandemic and thus used virtual methodology to maintain full social distancing. All participants were provided with full information and gave consent to participate in the study as well as consent and assent for adolescent participation.

### Consent for publication

Not applicable.

### Availability of data and materials

All study materials and anonymised data in the form of quotes are provided as supplementary files.
